# Undernutrition and associated factors among adult HIV/AIDS patients receiving antiretroviral therapy in eastern zone of Tigray, Northern Ethiopia: a cross-sectional study

**DOI:** 10.1186/s13690-020-00486-z

**Published:** 2020-10-15

**Authors:** Tsegu Hailu Gebru, Haftea Hagos Mekonen, Kbrom Gemechu Kiros

**Affiliations:** Department of Nursing, Adigrat University, Adigrat, Tigray Ethiopia

**Keywords:** Undernutrition, HIV/AIDS, Antiretroviral therapy, Tigray, Ethiopia

## Abstract

**Background:**

Undernutrition and HIV/AIDS are highly prevalent in sub-Saharan Africa, Ethiopia inclusive as linked in a vicious cycle. Thus, several studies have documented that undernutrition among HIV/AIDS patients increases the risk of mortality, decrease survival rates, affect the overall clinical outcome and quality of life.

Despite this fact, information about the burden of undernutrition and associated factors among adults receiving antiretroviral therapy is lacking in the particular study area. Hence, this study aimed to examine the prevalence of undernutrition and associated factors among adult HIV/AIADS patients receiving antiretroviral therapy patients in Eastern Zone of Tigray, Northern Ethiopia.

**Methods:**

A cross-sectional research design was adopted in data collection while systematic sampling technique was employed to sample and select the study subjects. A structured questionnaire was used to collect information from 394 study subjects through face to face method.

Also, data on demographics, laboratory and anthropometric variables were collected from each selected patients sampled.

The data collected were entered and analyzed using SPSS version 22.. Bivariate and multivariable logistic regression analysis with 95% confidence interval were used to find factors associated with undernutrition. The adjusted odds ratio was calculated to show the strength of the association. Variables with *p*-value of < 0.05 were considered statically significant.

**Results:**

The mean age of the respondents was 41 (± 10). Out of 394 study respondents, about 42.9% of them were undernourished (95% CI: 37.8–47.7).

Respondents who had CD4+ count less than 200 cells/μl (AOR = 1.84; 95% CI: 1–3.36), being advanced clinical staging (AOR = 3.6; 95% CI: 2.11–6.18), and not taking co-trimoxazole preventive therapy (AOR = 2.38; 95% CI: 1.21–4.6) were independently associated with undernutrition.

**Conclusion:**

The result of this study indicated that the prevalence of undernutrition was high.

Respondents with advanced clinical stage of CD4+ count less than 200 cells/ul and those that were not taking co-trimoxazole preventive therapy was found to be positively associated with undernutrition.

Therefore, the implementation of nutritional programs is very crucial to improve the nutritional status of HIV/AIDS patients in the particular study.

## Background

Human Immunodeficiency Virus (HIV) and AIDS remained a significant problem health burden particularly in developing countries.

By the end of 2016, about 34.5 million adults were living with HIV and an estimated 1 million patients died due to Acquired Immunodeficiency Syndrome (AIDS) related conditions [1]. Around 64% of the global HIV positive population are found in Sub-Saharan Africa (SSA) [1]. Globally, over 800 million people remain chronically undernourished and the HIV epidemic largely overlaps with populations already suffering from low diet quality and quantity [2].

Undernutrition is the predominant problem for HIV infected patients. It creates a vicious cycle which may catalyze progression from HIV infection to AIDS [3]. Poor nutrition resulting in weight loss, muscle wasting, weakness, nutrient deficiencies then it leads impaired immune system (poor ability to fight HIV and other infections, increased oxidative stress, increased vulnerability to infections). Hence, increased HIV replication, hastened disease progression and increased morbidity, then it leads to increased nutritional needs, and increased loss of nutrients. On the other hand, adequate dietary intake enhances the therapeutic effect of medicines, boosts the immune system (thus helping to fight against the disease and to maintain body weight), increases longevity, and promotes healthy living [4].

The relationship between HIV and undernutrition is bidirectional. Both are related each other in causing progressive damage to the immune system. HIV compromises nutritional status and poor nutrition further weakness the immune system of individuals.. The effect of malnutrition by itself can reduce the cluster of differentiation-four (CD4+) T cells and lead to abnormal B-cell response. For HIV infected patients, poor nutrition aggravates the effect of HIV by further decreasing the immune system [3, 5], thereby leading in undernutrition, increasing the risk of morbidity and mortality, and potentially reducing the efficacy of antiretroviral therapy (ART) [6].

Besides, HIV/AIDS patients often do not take enough food because of the illness and the ART medications decrease the appetite, change the taste of food and prevent the body from absorbing it. Similarly, the disease associated-conditions such as sore of the mouth, nausea, and vomiting make it challenge to eat [7]. A well-nourished people with HIV and who have a controlled viral load are more likely to be able to withstand the effect of HIV infection, supporting immune status and possibly delaying the progression of the disease [8]. Adequate nutrition is necessary for HIV infected patients to manage opportunistic infections, optimize response to medical treatment, keep up the immune system, and to support the ideal quality of life [2, 9].

Malnourished HIV positive people experience lower survival rates, affect the overall clinical outcome and quality of life [10].

Undernutrition among HIV infected clients is often preventable, but recovering is difficult particularly those who are wasted, need more protein, and micronutrient intake to increase a weak-ended immune system.

[11]. Antiretroviral therapy (ART) was thought to be a solution to HIV-associated undernutrition. It is well established that most of clinically undernourished people living with HIV who start ART will improve or stabilize their weight [12]. As HIV infection progresses, it causes a catabolic state and increased susceptibility to infection which are compounded by lack of caloric and leading to progressive worsening of undernutrition. Thus, HIV viral load predicted weight loss and it appears that ART has made a reduction in the prevalence of undernutrition probably through the reduction in viral load. Because ART result in reduced viral replication, improved immunologic function and dramatic improvements in health, and prevents undernutrition [2, 13].

But, undernutrition remains a big issue for patients taking ART [14].

The prevalence of undernutrition among HIV-infected individuals was 19% in Tanzania [15] and 10% in Zimbabwe [16]. Ethiopia is also one of the countries hit hardest by the HIV epidemic alongside malnutrition [17]. The prevalence of undernutrition among HIV/AIDS patients receiving ART in Ethiopia ranges from 12.3 to 46.8% [18–24].

Different studies in different parts of the world revealed the factors associated with undernutrition were age, poor medication adherence, anemia, duration of the disease, opportunistic infection, advanced stage WHO, low CD4+ count, not taking co-trimoxazole, current substance use, marital status, residence, and active tuberculosis (TB) [18, 19, 21–25]. But the above factors are different across the studies.

Besides remarkable efforts are made in increasing the treatment coverage of HIV/AIDS in the past decades, the high burden of HIV/AIDS and undernutrition has remained the major problems of health care systems in SSA [26].

Even though Ethiopia has significantly improved the treatment coverage of HIV/AIDS (71%) in 2017 [27], HIV/AIDS prevalence remained high (0.9%) [28]. Ethiopia is one among the 25 countries worldwide and 17 countries in Africa region with the highest number of new HIV infections [27]. HIV/AIDS and undernutrition combine to emasculate the immunity of many Ethiopians [29]. HIV related debilitating infections have severe nutritional consequences that commonly precipitate weight loss and finally lead to a wasting syndrome [30]. The high rates of undernutrition in Ethiopia worsens the impact of HIV and pose significant challenges to HIV care and treatment programs [31]. This calls for attention in improving the treatment effectiveness of existing HIV/AIDS services and one of the focus area of this actions was strengthening the nutritional assessment HIV/AIDS patients [20].

Continued study of the relationship between malnutrition and ART is important in low-income countries like Ethiopia, where many HIV/AIDS patients lack access to sufficient quantities of nutritious foods, which poses additional challenges to the success of ART [2, 6]. New information concerning the burden of undernutrition among HIV positive patients is very important to provide quality care. Despite this fact, there was a limited study indicated the burden of undernutrition and associated factors among HIV infected patients in Eastern Zone in particular and in Tigray region in general. Hence, this study aimed to assess the prevalence of undernutrition and associated factors among adult HIV/AIADS patients receiving ART in Eastern Zone of Tigray, Northern Ethiopia.

## Methods

### Study area, design, and period

The study was conducted in public health facilities of Eastern Zone, which is found in Tigray region, Ethiopia. Eastern Zone is located in the Northern part of Ethiopia, which is 888 km away from the capital City Addis Ababa. Based on the 2007 census conducted by central static agency of Ethiopia, this Zone has a total population of 755,343 [32]. There are seven primaries and two general hospitals that provide ART services in Eastern Zone Tigray [33]. Out of these, three primaries and the two general hospitals were selected randomly. The study hospitals were Adigrat general hospital, Wukro general hospital, Atsbi primary hospital, Hawzen primary hospital, and Fatsi primary hospital.

The research study was an institutional-based as it employed cross-sectional research design conducted from April to May for two consecutive months in 2019.

### Population

The study population were people living with HIV/AIDS that has been enrolled for ART follow-up service within the age cohort of greater than or equal to 18 years old as they are currently receiving ART treatment.

However, study respondents who are pregnant women, lactating mothers (6 months of postpartum), patients with edema like in congestive heart failure and ascites, and critically ill and/or patients with spinal deformity were excluded.

### Sample size determination and sampling technique

The required sample size was determined using single population proportion formula taking 36.8% ~ 0.37 prevalence of undernutrition among adults with HIV/AIDS on ART with 5% of margin error and 95% confidence interval (CI) [20]. By adding 10% of non-response rate, the final sample size was determined as 394. To select the required sample size the total sample size was proportionally allocated to the five public hospitals. Accordingly, the list of the patients were taken from the follow-up unit of the five public hospitals, and the sampling frame was developed. Then the first study subject was randomly selected from the sampling frame by using the lottery method and those participants were selected using a systematic sampling technique from the sampling frame.

### Data collection instruments and procedures

Data were gathered using a structured and interviewer-administered questionnaire with the chart/document review (Additional file 2). The questionnaire consisted of four parts. Part I: Sociodemographic characteristics (gender, age, marital status, educational level, occupation, residence, and average house hold monthly income). Part II: Lifestyle/behavioral characteristics such as alcohol consumption, khat chewing and smoking status. Part III: ART medication adherence. Part IV: Chart/document review used to extract information related to HIV related characteristics, including immunological status and clinical characteristics (world health organization clinical stage, CD4 count, functional status, and opportunistic infections). The anemia status of the participants was ascertained using the standard criteria for female and male adults. Accordingly, patients were graded anemic when the hemoglobin concentration was less than 12.0 g/dl and less than 13.0 g/dl for females and males respectively.

Anthropometric measurement was carried out to determine the nutritional status of the study participants’ by using Body Mass Index (BMI). The weight of the study subjects was measured using a beam balance to the nearest 0.1 Kg and measuring range up to 160 Kg. Weight was measured with light clothing and no shoes. Calibration was performed before weighing each participant by setting it to zero. Weighing scale also checked against a standard weight for its accuracy on a daily basis. The height of the respondents was measured using a vertical height scale standing upright in the middle of the board and recorded to the nearest 0.5 cm. Respondents were asked to take off their shoes, stand erect, and look straight in the horizontal plane. The occiput, shoulder, buttocks, and heels touched measuring board and height was recorded to the nearest 0.01 cm [34]. Then BMI was computed by dividing weight in kg by the square of the height in meters (kg/m2). Accordingly, the study participants were classified as undernourished (underweight) if their BMI was less than 18.5 kg/m2.

To maintain consistency, the questionnaire was first prepared in English then translated to the local language (Tigrigna) and was back-translated to English by professional translators. Five individuals who have completed their BSC in nursing education from a recognized University were recruited as data collectors. Another two senior BSC holder health officers who have experience in data collection were assigned as supervisors to check for the daily activity, consistency, and completeness of the questionnaire and to give appropriate support during the data collection process.

### Data processing and analysis

The collected data were entered and analyzed using SPSS version 22. Both bivariate and multivariable logistic regression analysis were used to examine the association between each independent variable and dependent variable. The bivariate analysis is used to filter the important predictors. For that purpose, we have used a cut point of *p*-value < 0.25 significance level in the bivariate logistic regression to transfer variables to multivariate analysis. The role of multivariate logistic regression was to determine statistical significance of the independent variables with the outcome variable and to show the strength of association between a set of independent variables and the outcome variable. The *p*-value < 0.05 was used to test for association to show statistical significance in the multivariate logistic regression while the odds ratio (OR) was estimated at 95% CI to show the strength of association.

Finally, text and tables were used to describe the results.

### Definition of variables

Medication adherence was assessed using Morisky medication adherence score to ART medications having eight questions each with yes = 1 and no = 0, good adherence if they score 7–8, and poor adherence if they score ≤ 6 [35].

Alcoholic - a person who drinks 10.5 units of alcohol and above per week [36].

## Results

### Socio-demographic characteristics of respondents

A total of 394 HIV positive patients were included. Of these, 249 (63.2%) of the patients were females. The mean age (± SD) of the respondents was 41 (± 10). Concerning the marital status of the study population, 126 (32%) of the respondents were married. On the other hand, 207 (52.5%) of the respondents were living in urban areas (Table 1).

**Table 1:** Socio-demographic characteristics of HIV-infected patients in Eastern Zone Tigray, Ethiopia, 2019

Variables	Category	Frequency	Percentage
Gender	Male	145	36.8
	Female	249	63.2
Age	18–24	23	5.8
	25–34	64	16.2
	35–44	157	39.8
	≥45	150	38.1
Marital status	Married	126	32
	Single	81	20.6
	Divorced	106	26.9
	Widowed	81	20.6
Level of education	Can’t read and write	194	49.2
	Can read and Write	80	20.3
	Primary school	69	17.5
	Secondary school	36	9.1
	Diploma and above	15	3.8
Occupation	Farmer	139	35.3
	Housewife	44	11.2
	Governmental employee	35	8.9
	Daily worker	49	12.4
	Merchant	37	9.4
	No work	83	21.1
	Other	7	1.8
Religion	Orthodox	381	96.7
	Muslim	6	1.5
	Catholic	6	1.5
	Other	1	0.3
Ethnicity	Tigray	392	99.5
	Amhara	2	0.5
Residence	Urban	207	52.5
	Rural	187	47.5
House owner	No	160	40.6
	Yes	234	59.4
Family size	≤ 3	174	44.2
	4–6	196	49.7
	> 6	24	6.1
Monthly income	< 193	16	4.1
	193–420	77	19.5
	421–1700	200	50.8
	> 1700	101	25.6

### Nutritional status, clinical, behavioral, and immunological characteristics of respondents

Prevalence of undernutrition (BMI ≤ 18.5 kg/m^2^) was 42.9%. Of those, 26.6 and 16.2% of the study subjects had moderate (BMI 16–18.5 kg/m^2^) and severe (BMI < 16 kg/m2) malnutrition respectively. Concerning medication adherence, about 365(92.6%) of participants had poor adherence to the ART medication. The clinical condition of the participants at ART initiation indicated that about 205(52%) had an opportunistic infection. Most patients started ART with advanced disease. For instance, 257(65.2%) of the study participants had baseline clinical stage II and above. On the other hand, 55(14%) of the respondents did not use ionized preventive prophylaxis (IPT) (Table 2).

**Table 2:** Behavioral, clinical, and nutritional status of HIV-infected patients in Eastern zone of Tigray, Ethiopia, 2019

Variables	Category	Frequency	percentage
Khat chewing status	No	384	97.5
	Yes	10	2.5
History of smoking	No	380	96.4
	Yes	14	3.6
Alcohol consumption	No	327	83
	Yes	67	17
Undernutrition	Yes	169	42.9
	No	225	57.1
Medication adherence	poor	365	92.6
	Good	29	7.4
TB status	No	270	68.5
	Yes	124	31.5
Functional status	Working	255	64.7
	Ambulatory	77	19.5
	Bedridden	62	15.7
Opportunistic infection	No	189	48
	Yes	205	52
CD4+ count (cells/μl)	< 200	92	23.4
	200–350	66	16.8
	351–500	87	22.1
	> 500	146	37.8
Anemia	Yes	169	42.9
	No	225	57.1
WHO clinical stage	Stage I	137	34.8
	stage II and above	257	65.2
IPT prophylaxis	No	55	14
	Yes	339	86
Co-tromoxazole Prophylaxis	No	58	14.7
	Yes	336	85.3
Duration of HIV/AIDS	< 2 years	13	3.3
	≥ 2 Years	381	96.7

### Bivariate and multivariable logistic regression for factors associated with undernutrition among ART initiated HIV/AIDS patients

In this study, both bivariate and multivariable logistic regression analysis were computed. In the bivariate logistic regression, marital status, educational level, family size, CD4+ count, WHO clinical staging, active TB, co-trimoxazole therapy, and functional status were identified as candidate variables for multivariable logistic regression analysis. Out of those, only three variables were significantly associated with undernutrition. Therefore, participants with CD4+ count < 200 cells/μl (AOR = 1.84; 95% CI: 1–3.36), WHO clinical stage II and above (AOR = 3.6; 95% CI: 2.11–6.18), and not taking co-trimoxazole prophylaxis (AOR = 2.38; 95% CI: 1.21–4.67) were more likely to be at risk to develop undernutrition (Table 3).

**Table 3:** Bivariate and multivariable analysis of factors associated with undernutrition in Eastern Zone, Tigray, Ethiopia, 2019

	Undernutrition			
Variables	Yes	No	COR (95% CI)	AOR (95% CI)
	N^o^ (%)	N^o^ (%)		
**Marital status**				
Married	56 (33.1)	70 (31.1)	1	1
Single	30 (17.8)	51 (22.7)	1.36(.768–2.41)	1.101(.58–2.09)
Divorced	53 (31.4)	53 (23.6)	0.8(.477–1.343)	.88(.488–1.587)
Widowed	30 (17.8)	51 (22.7)	1.36(.768–2.41)	1.14(.585–2.221)
**Educational level**				
Cannot read and write	76 (45)	118 (52.4)	1.774(.618–5.094)	.94(.293–3.037)
Can read and write	26 (15.4)	54 (24)	2.37(.777–7.254)	1.68(.5–5.64)
Primary school	34 (20.1)	35 (15.6)	1.17(.384–3.60)	.895(.265–3.015)
Secondary school	25 (14.8)	11 (4.9)	0.50(.146–1.734)	.43(.114–1.636)
Diploma and above	8 (4.7)	7 (3.1)	1	1
**Family size**				
≤ 3	86 (50.9)	88 (39.1)	1	1
4–6	73 (43.2)	123 (54.7)	1.647 (1.087–2.493)	1.21(.75–1.95)
> 6	10 (5.9)	14 (6.2)	1.368(.577–3.247)	1.23(.47–3.22)
**CD4+ cells/μl counts**				
< 200	31 (18.3)	61 (27.1)	2.221 (1.296–3.807)	1.84 (1–3.36)*
200–350	24 (14.2)	42 (18.7)	1.975 (1.088–3.584)	1.53(.79–2.95)
351–500	35 (20.7)	52 (23.1)	1.677(.981–2.865)	1.5(.83–2.70)
> 500	79 (46.7)	70 (31.1)	1	1
**WHO Clinical staging**				
Stage I	87 (51.5)	50 (22.2)	1	1
Stage II and above	82 (48.5)	175 (77.8)	3.713 (2.4–5.74)	3.6 (2.11–6.18)*
**Active TB status**				
No	122 (72.2)	148 (65.8)	1	1
Yes	47 (27.8)	77 (34.2)	1.35(.874–2.086)	.66(.39–1.1)
**Co-trimoxazole**				
No	17 (10.1)	41 (18.2)	1.992 (1.1–3.65)	2.38 (1.21–4.67)*
Yes	152 (89.9)	184 (81.8)	1	1
**Functional status**				
Working	99 (58.6)	156 (69.3)	1	1
Ambulatory	36 (21.3)	41 (18.2)	.723(.432–1.208)	1.08(.56–2.07)
Bedridden	34 (20.1)	28 (12.4)	.523(.299–.915)	.87(.43–1.76)

**Note:** a. 1 = Reference category, b. * = statistically associated variable in multivariable logistic regression at *P* < 0.05

## Discussion

This study is focused on assessing the prevalence of undernutrition and associated factors among HIV infected patients after the initiation of ART. The overall prevalence of undernutrition was 42.9% (95% CI, 37.8–47.7). This result is in line with other institutional-based studies conducted in Ethiopia such as Humora hospital (42.3%) [37] and Jima University specialized hospital (46.8%) [24]. But this is higher than other studies done in Tanzania (19%) [15] and Zimbabwe (10%) [16]. Similarly, it is also higher than other studies done in Ethiopia such as in Arba Minch Town (23.2%) [21], 12.3% in Dilla University [19], and 23.2% in Dembia district [23].

This different result among different studies could be explained by the discrepancy in health care awareness of the community and the sample size difference. Or it could be due to the difference in socio-economic and feeding styles of different ethnic groups of the country. Besides, it might be due to the difference and improvements in healthcare services. Or it might be as a result of the patients reporting with advanced stage of HIV infection to medical facilities.

This study indicated that CD4 + -T cell count had a strong positive association with nutritional status. Participants with CD4+ <  200 cell/μl had 1.84 times chance of being undernourished compared to those with CD4+ cells/μl count > 500 (AOR = 1.84; 95% CI: 1–3.36). Other studies done in Ethiopia [19, 21, 23, 24] showed similar associations. This result is also in line with other studies done in developed and developing countries [10, 38–40]. This relation is explained by the association of a low CD4 cell count with more advanced stages of infection, implying a higher frequency of opportunistic infections, which affect nutritional status by increasing metabolism and diminishing food intake because of factors such as fever, oral and esophageal lesions, anorexia and diarrhea [41].

Participants with world health organization clinical stage II and above were found 3.6 times more likelihood of being undernourished compared to those with WHO clinical stage I (AOR = 3.6; 95% CI: 2.11–6.18). This is also comparable with other studies that showed similar results in Ethiopia [19, 21, 22, 24]. This might be because patients with advanced disease stages are more susceptible to develop comorbid conditions. Thus, additional management of such comorbid conditions and the regular ART treatment worsen the side effects like loss of appetite and poor nutritional status of the patient, which compromise their resistance to the disease.

The initiation of co-trimoxazole preventive therapy at the start of ART was associated with the nutritional status of the participants. Based on this study, the odds of developing undernutrition among patients receiving ART who did not start co-trimoxazole preventive therapy were 2.38 times more likely to develop undernutrition compared to those who were started co-trimoxazole preventive therapy (AOR = 2.38; 95% CI: 1.21–4.67). The finding of this research is consistent with the studies conducted in Nigeria [42] and public health facilities of Arba Minch Town [21]. This similarity might be due to the fact antimicrobial effect of co-trimoxazole on bacterial disease and other opportunistic infections can help to improve the overall status of the patients.

### Limitations of the study

The cross-sectional nature of the study limits the investigation to the level of the association between determinants and outcomes of interest (undernutrition). On the other hand, not using comprehensive anthropometric measurement tools like hip circumference limits this study because, only using BMI may not the exact measurement of malnutrition.

## Conclusion

The result of this study indicated that the prevalence of undernutrition was high. Furthermore, being an advanced clinical staging, having a low CD4+ count, and not taking co-trimoxazole preventive therapy was positively associated with undernutrition. Therefore, the implementation of nutrition programs for patients living with HIV is crucial. Especially, the intervention must be focused for those patients who have advanced clinical stage of CD4+ count less than 200 cells/ul. Also, the emphasis must be considered for patients to undergo co-trimoxazole preventive therapy. Moreover, effort should be taken by governmental and non-governmental stakeholders to improve the nutritional status of patients under ART to increase treatment effectiveness.

## Supplementary information

**Additional file 1.** S1 dataset- SPSS data of the questionnaire results.

**Additional file 2.** S1 Appendix- English and Tigrigna version of the questionnaire.

## Data Availability

The dataset used and/or analyzed during the current study is included with in the article’s additional file (Additional file 1).

## Abbreviations

AIDS: Acquired immune Deficiency Syndrome
AOR: Adjusted Odds Ratio
ART: Antiretroviral Therapy
BMI: Body Mass Index
COR: Crude Odds Ratio
CI: Confidence Interval
HIV: Human immunodeficiency Virus
IPT: Isoniazid preventive therapy
WHO: World Health Organization
SPSS: Statistical Package for Social Science
