# Supplementary material for: Undernutrition and associated factors among adult HIV/AIDS patients receiving antiretroviral therapy in eastern zone of Tigray, Northern Ethiopia: a cross-sectional study
Source: Arch Public Health. 2020 Oct 15;78:100. doi: 10.1186/s13690-020-00486-z (PMC7559062; doi:10.1186/s13690-020-00486-z)
Supplement: Supplementary file 2 — Additional file 2. S1 Appendix- English and Tigrigna version of the questionnaire. [file 13690_2020_486_MOESM2_ESM.docx]

**English version of data collection tools**

**English version information sheet and consent form**

**Information sheet**

**Name of principal Investigator**: Tsegu Hailu

**Name of organization**: University of Adigrat College of medicine and Health science and department of nursing.

**Introduction**: This information sheet & consent form is prepared by the principal investigator whose aim is to study assessment of undernutrition for HIV/AIDS on ART treatment and associated factors. Others that will be participated in this research include two trained senior health officer supervisors, and five Nurses for data collection.

**Purpose of the research project**: Assessment of undernutrition and its associated factors among adult HIV/AIADS patients receiving ART in Eastern zone of Tigray, Northern Ethiopia.

**Procedure**: This study will be conducted on HIV/AIDS patients who are on ART. Before starting the research, Ethical clearance will be obtained from university of Adigrat ethical review board and a permission letter from Tigray Regional Health Bureau, Eastern Zone Health office, Medical directors of each hospitals, and written consent from the study participants will also be obtained. At last, ART patient will be interviewed with structured questioner

Risk and or discomfort: by participating in this research project you will not feel any discomfort and the interview will last only 20 to 30 minutes. Information gathered will be kept confidently. There is no risk at all by participating in this research.

Benefits: your participation in this research project, may not give you a direct benefit. But it has great role to achieve improvements in nutritional status of HIV/AIDS patients.

Incentives: you will not get paid or have any other incentives to take part in this project.

**Confidentiality and anonymity:** The information we collate form this research will be kept confidential. Information about you that will be collected from this study will be stored in a file, which will not have your name on it. The investigator will store by locking so that unintended body will not have accesses to it. Your name will not be written on this form & used in connection with any of the information you tell me.

Right to refuse and/ withdraw: you have full right to refuse form participating in this research you can choose not to respond some or all of the questions. This will not affect your health service delivery that you got form the hospital.

## Consent form:

Hello! My name is ______________________I am working in the research team as an academic researcher in the University of Adigrat, College of Medicine and Health Science, and department of nursing. I would like to gather information regarding the nutritional status of HIV/AIDS patients. While you are in this health institution which is very important to improve the service delivery and nutritional status. Your cooperation and willingness to give the information is helpful in identifying problems related to the concern. We have identified you as a study participant believing that you would be willing to respond to questions to give me your sincere and truthful answers. By participating in this research project, your name or address will never be written in this form. All information that you give will be kept strictly confidential. Your participation is voluntary and you are not obliged to answer any question you do not wish to answer. If you have any question or anything unclear, please feel free to ask. If you are not still comfortable along the discussion process you have full right to drop it any time you want. You will not be asked to give reasons and you will not miss any chance of service delivery from the hospital for doing so.

If you are clear with the information provided, do I have your permission to continue?

1. If yes_______________ thank you, continue to the next page.
2. If no________________ thank you, stop.

# **Structured English Version Questionnaire**

## University of Adigrat

## College of Medicine and Health Sciences

## Nursing Department

Questionnaire developed for data collection on undernutrition among HIV/AIDS patients receiving antiretroviral therapy in Eastern Zone, Tigray, 2019

### Identification:

Name of the health institution_______________________ Code No.___________

Date of interview________________ time started ________time finished_______

Interviewer or supervisor who certified that, informed consent has been given from the respondents.

Name of interviewer _________________________ Signature ____________

Name of supervisor __________________________ signature _____________

**Part I: Socio-demographic Characteristics**

| **S.N^o^** | **Variables** | **Categories** | **Remark** |
| --- | --- | --- | --- |
| 101 | Age in years | _______________ |  |
| 102 | Gender | 1. male 2. female |  |
| 103 | Marital status | 1. married 2. single 3. Divorced 4. Widowed |  |
| 104 | What level of education do you accomplish? | 1. Can’t read and write 2. Can read and write 3. Primary school 4. Secondary school 5. Diploma and above |  |
| 105 | What is your occupation? | 1. Farmer 2. House wife 3. Governmental employee 4. Daily worker 5. Merchant 6. No work 7. Other/specify__________ |  |
| 106 | Number of people in house hold | _______ |  |
| 107 | Where do you live? | 1. Rural 2. Urban |  |
| 108 | Do you have your own house? | 1. Yes 2. No |  |
| 109 | Average monthly income/Ethiopian birr | ___________________ |  |
| 110 | What is your religion? | 1. Orthodox 2. Muslim 3. Protestant 4. Other /specify______ |  |
| 111 | Where is your region/Ethnicity? | 1. Tigrigna 2. Amhara 3. Afar 4. Other /specify_____ |  |

**Part II: lifestyle/behavioral characteristics of respondents**

| **S. No** | **questions** | **Response** |
| --- | --- | --- |
| **112** | Have you ever tried to chewing khat? | 1. Yes 2. No |
| **113** | Have you ever smoked cigarette, | 1. Yes 2. No |
| **114** | Have you ever drunk alcohol? | 1. Yes 2. No |

**Part III: ART Medication adherence**

| 115 | \| Do you sometimes forget to take your pills? \| \| --- \| | Yes | No |
| --- | --- | --- | --- | --- |
| 116 | People sometimes miss taking their medications for reasons other than forgetting. Thinking over the past two weeks, were there any days when you did not take your medicine? |  |  |
| 117 | Have you ever cut back or stopped taking your medicine without telling your doctor because you felt worse when you took it? |  |  |
| 118 | When you travel or leave home, do you sometimes forget to bring along your medicine? |  |  |
| 119 | Did you take all your medicine yesterday? |  |  |
| 120 | When you feel like your symptoms are under control, do you sometimes stop taking your medicine? |  |  |
| 121 | Taking medicine every day is a real inconvenience for some people. Do you ever feel hassled about sticking to your treatment plan? |  |  |
| 122 | How often do you have difficulty remembering to take all your medicine?  a. Never/rarely  b. Once in a while  c. Sometimes  d. Usually  e. All the time |  |  |

| **Part-IV: Base line (at ART initiation) clinical, laboratory and ART information (Filed from ART registration book/ interview)** |
| --- |

|  | **Variables** | **Response** | Remark |
| --- | --- | --- | --- |
| 123 | \| Duration since diagnosis of HIV/AIDS \| \| --- \| | ______Years  _______Month |  |
| 124 | Functional status | 1. Working 2. Ambulatory 3. Bedridden |  |
| 125 | Opportunistic infection/last 6 months | 1. Yes 2. No |  |
| 126 | Weight | _______________ |  |
| 127 | Height | ____________ |  |
| 128 | Hemoglobin level | _______________ |  |
| 129 | CD4+ count | ______________ |  |
| 130 | WHO clinical stage | 1. Stage I 2. Stage II 3. Stage III 4. Stage IV |  |
| 131 | Isoniazid preventive therapy (IPT) | 1. Yes 2. No |  |
| 132 | Co-trimoxazole prophylaxis | 1. Yes 2. No |  |
| 133 | Is there current TB? | 1. Yes 2. No |  |

**ናይ ተሳተፍቲ መረዳእታ መሰብሰቢ ቅጥዒ ብ ትግርኛ አብ ዓዲግራት ዩኒቨርሲቲ ጥዕና ሳይንስ ኮለጅ ነርሲንግ ትምህርቲ ክፍሊ**

Code __________________

Date___________________

MRN_____________________

እዚ መረዳእታ መአከቢ ዕላሙኡ አብ ምብራቓዊ ዞባ ዝርከቡ መንግስታዊ ሆስፒታላት ውሽጢ ፀረ-ኤችአይቪ መድሓኒት ምስጀመሩ ሕፅረት ምግቢ ንክክሰት ምክንያት/ መንቀሊ ዝኮኑ ነገራትን በዝሖም ንምፍላጥ እንትኮን ፅንዓቱ ዝከናወን/ዝካየድ ብቃለመጠይቅን ካብ ሕክምና ካርድ መረዳእታ ብምውሳድን እዩ ፡፡ እዚ ፅንዓት ናይ መረዳእታ ክፍተት ንምምላእን መሰረታዊ መረዳእታ ንምርካብን ፥ ን ፕሮግራም መውፃእቲ፣ ን ውሳነ ወሃብትን ን ምግቢ-ኤችኣይቪ ሕክምና ኣፈፀምቲ መረዳእታ ንክረክቡ የክእል፡፡

**ምስጢር ምሕላው**

ዝጥይቆም/ን ሕቶታት በቐሊሉ ክመልሰዎም ዝክእሉ እዮም ስለዚ ሓሳቦም ብነፃነት ምግላፅ ንክክእሉ ስሞም ምፅሓፍ ኣየድልን፡፡ነገር ግን ስለ ናቶም/ን ሓቀኛ ዝኮነ መልሲ ኣድላዪ እዩ፡፡ ንዝህብዎ መረዳእታ/ሓበሬታ ሚስጥራዊነቱ ዝተሓለወ እዩ፡፡እዚ ቃለ መሕትት ዝምልኣሎም ፍቓደኛታት ንዝኮኑ ሰባት ጥራሕ እዩ፡፡ንምስታፍ ፍቓደኛ እንተዘይኮይኖም/ነን ኣብ ዝኮነ ግዘ ሙቁራፅ ትክእሉ ኢኩም/ክን፡፡ንዝህቡና ሓበሬታ/መረዳእታ ካብ ልቢ ነምስግን፡፡ ፅንዓቱ ዝተመልከተ ሕቶ እንተሃልይዎም በዚ ስልኪ ቁፅሪ ናብ ፀጉ ሃይሉ ደዊሎም ምጥያቕ ይክእሉ/ላ፡፡ ስልክ፡-251-914398661 ወይ ብኢሜል፡ [tsegshhailu16@gmail.com](mailto:tsegshhailu16@gmail.com) **ናይ ስምምዕነት ውዕል**

እዚ ፅንዓት ዘፅንዖ ግለሰብ ብዘረደአኒ መሰረት ብፍቓደይ ኣብዚ ፅንዓት ንምስታፍ

ፍቃደኛ እየ ፍቃደኛ ኣይኮንኩን ናይ ሓበሬታ ኣካቢ ሽም____________________ ፊርማ: __________ ዕለት--------

**መምርሒ፡ ናይ ተሳታፋይ/ተጠያቒ መልሲ ክቢ ( ) ብምግባር የቕምጡ ክፍሊ1: ናይ ተጠያቒ ማሕበራውን ዲሞግራፍያውን ኩነታት/ማሕበራዊ መረዳእታ ቃለ-መሕትት**

| **ተቁ** | **ሕቶታት/variables** | **መልሲ/categories** | | | | **ርኢቶ** |
| --- | --- | --- | --- | --- | --- | --- |
| 101 | ዕድመ | _________ ዓመት | | | |  |
| 102 | ፆታ | 1.ተባ 2. ኣንስታይ | | | |  |
| 103 | ኩነታት ሓዳርኩም/ን | 1. ዝተመርዐወ/ት 2. ዘይተመርዐወ/ት 3. ዝተፋትሐ/ት 4. ሰብኣያ ዝሞታ/ሰበይቱ ዝሞቶቶ | | | |  |
| 104 | ደረጃ ት/ቲ | 1. ዘየንብብን ዘይፅሕፍን 2. ዘንብብን ዝፅሕፍን 3. 1ይ ብረኪ 4. 2ይ ብርኪ 5. ዲፕሎማን ልዕሊኡን | | | |  |
| 105 | ስርሖም/ን | 1.ሓረስታይ 2. ናይ ገዛ ሰራሕተኛ 3. ናይ መንግስቲ ሰራሕተኛ 4.መዓልታዊ ሰራሕተኛ 5. ነጋዳይ 6. ስራሕ ዘይብሉ/ላ  7. ካሊእ (ይገለፅ)----------------------- | | | |  |
| 106 | ኣብ ሓደ ገዛ ውሽጢ ክንደይ ሰብ ትነብሩ (በዝሒ ስድራ) | ---------------------- | | | |  |
| 107 | ዝነብርሉ ቦታ | 1. ገጠር 2. ከተማ | | | |  |
| 108 | ኣብ ናይመን ገዛ ትነብሩ/ራ? | 1. ኣብ ናይ ባዕለይ 2. ኣብ ክራይ | | | |  |
| 109 | ወርሓዊ እቶቶም/ን ብማእከላይ ክንደይ ይከውን(ብገንዘብ) | ___________________ | | | |  |
| 110 | ሃይማኖትኩም/ን | 1. ኦርቶዶክስ 2. ሙስሊም 3. ካቶሊክ 4. ካሊእ (ይጠቐስ)_____ | | | |  |
| 111 | ብሔር | 1.ትግራይ 2. ኣምሓራ 3.ዓፋር 4. ካሊእ ይጠቐስ______ | | | |  |
|  | **ክፍሊ 2፡ባህርያታት/ፀባያት ሕሙም ዝምልከት** | | | | |  |
| 112 | ጫት ቕሒምኩም/ን ዶ ትፈልጡ/ጣ? | 1. እወ 2. ቅሒመ የፈልጥን | | | |  |
| 113 | ሽጋራ ዶ ኣትኪክካ/ኪ ትፈልጥ/ጢ? | 1.እወ  2. የፈልጥን | | | |  |
| 114 | አልኮላዊ መስተ ትሰትዩ/ያ ዶ? | 1. እወ 2. የሰትን | | | |  |
|  | **ክፍሊ3፡ ናይ ኤች አይቪ** **ኣጠቓቕማ መድሓኒት ዝምልከት** | | | **እወ** | **ኣይፋሉ** |  |
| 115 | \| ሓደ ሓደ ግዘ መድሓኒትኩም ምውሳድ ረሲዕኩም/ን ዶ ትፈልጡ/ጣ? \| \| --- \| | | |  |  |  |
| 116 | ሰባት ሓደ ሓደ ግዘ ብምርሳዕ ዘይኮነስ ብካሊእ ምክንያት መድሓኒት ምውሳድ የቃርፁ እዮም፡፡ ንዝሓለፉ ክልተ ሰሙናት ብምስትዋስ መድሓኒት ዘይወሰድሎም/ዳሎም ማዓልትታት ኣለዉ ዶ? | | |  |  |  |
| 117 | ብዘይ ናይ ዶክተር ትእዛዝ መድሓኒት እንትወሰዱ/ዳ ምልክታት እቲ ሕማም ዝባኣሱ እንትመስሉ መድሓኒት ምውሳድ ኣቓሪፆም/ን ዶ ይፈልጡ/ጣ ? | | |  |  |  |
| 118 | ሓደ ሓደ ግዘ መገሻ /ካብ ገዘኦም እንትወፅኡ መድሓኒት ምውሳድ ረሲዖም/ን ዶ ይፈልጡ/ጣ? | | |  |  |  |
| 119 | ትማሊ ኩሉ መድሓኒቶም/ን ዶ ወሲዶም/ን? | | |  |  |  |
| 120 | ምልክታት እቲ ሕማም ዝጠፍኡ እንትመስሉ መድሓኒት ምውሳድ ኣቓሪፆም/ን ዶ ይፈልጡ/ጣ? | | |  |  |  |
| 121 | መድሓኒት ኩሉ ግዘ ምውሳድ ንዝተወሰኑ ሰባት የሰልችዮም እዩ ንሶም/ን መድሓኒት ምውሳድ ሰልቺኩም ፕሮግራምኩም/ን ኣዛቢዕኩም ዶ ትፈልጡ? | | |  |  |  |
| 122 | መድሓኒቶም/ን ንምውሳድ ምዝካር ይሽገሩ/ራ ዶ?   1. ብፍፁም 2. ሓሓሊፉ 3. መብዛሕቲኡ ግዘ 4. ኩሉ ግዘ | | |  |  |  |
|  | **ክፍሊ4፡ መበገሲ(ናይ ፀረ-ኤች አይቪ መድሓኒት እንትጀምሩ) ዝነበሩ ናይ ላብራቶሪ ና ሕክምና መረዳእታ (ካብ ፀረ- ኤች አይቪ መድሓኒት መከታተሊ መዝገብን ካብ ሕሙም ብምጥያቕን ዝምላእ)** | | | | |  |
| 123 | HIV/AIDS ምህላዎም/ን ካብ ዝፈልጡ/ሕክምና ካብ ዝጅምሩ ክንደይጌሮም/ን | | _____ ዓመት  ______ወርሒ | | |  |
| 124 | ናይ ሕማም አጠቓላሊ ኩነታት (ART እንትጅምር) | | 1. ስራሕ ምስራሕ ዝክእል 2. ምንቅስቓስ ዝክእል 3. ናይ ኣልጋ ቁራኛ | | |  |
| 125 | ካሊእ ኦፖርትኒቲክ ኢንፈክሽን (IOs) እንተነይሩ (ART እንትጅምር) | | 1. ኣለ 2. የለን | | |  |
| 126 | ክብደት (ART እንትጅምር) | | ___________________ | | |  |
| 127 | ቁመት( ART እንትጅምር) | | ___________________ | | |  |
| 128 | ናይ CD4 ቁፅሪ (ART እንትጅምር) | | ________________ | | |  |
| 129 | ናይ ሄሞግሎቢን መጠን (ART እንትጅምር) | | ________________ | | |  |
| 130 | WHO ክሊኒካል ደረጃ (ART እንትጅምር) | | 1. ደረጃ I 2. ደረጃ II 3. ደረጃ III 4. ደረጃ IV | | |  |
| 131 | ናይ ቲቢ መከላከሊ መድሓኒት (INH) ወሲዶም/ን ዶ ነይሮም? | | 1. እወ 2. ኣይወሰድኩን | | |  |
| 132 | ኮትሪሞክዛዞል ይወስዱ/ዳ ዶ ነይሮም/ን? | | 1. እወ 2. ኣይወሰዱን | | |  |
| 133 | ሐዚ ናይ ቲቢ ሕማም ኣሎ ዶ? | | 1. እወ 2. የለን | | |  |
